# Successful closure of a refractory giant (15 sq mm) macular hole with amniotic membrane graft

**DOI:** 10.1093/jscr/rjae013

**Published:** 2024-01-30

**Authors:** Kakarla Venkata Chalam, Marib Akanda, Meenakshi Subramanian

**Affiliations:** Department of Ophthalmology, Loma Linda University School of Medicine, 11370 Anderson St., Suite 1800, Loma Linda, CA 92354, United States; Department of Ophthalmology, Loma Linda University School of Medicine, 11370 Anderson St., Suite 1800, Loma Linda, CA 92354, United States; Department of Ophthalmology, Loma Linda University School of Medicine, 11370 Anderson St., Suite 1800, Loma Linda, CA 92354, United States

**Keywords:** large macular hole, amniotic membrane graft, pars plana vitrectomy, visual recovery, surgery, treatment

## Abstract

The management of macular hole defects has undergone a significant transformation with the advent of advanced diagnostic tools and surgical techniques. These developments have enabled the effective treatment of macular holes that were previously considered untreatable. Although the majority of patients exhibit a positive response to initial treatment, a subset of patients may develop refractory macular holes that necessitate multiple surgeries for closure. In these instances, the utilization of amniotic membrane grafts to aid in the closure of large retinal holes presents a promising alternative. This report details the successful closure of a refractory giant macular hole (15 sq. mm) in a patient using an amniotic membrane graft, with improvement in visual acuity.

## Introduction

Macular hole is a debilitating ocular condition that affects the central region of the retina, termed the macula. The macula is responsible for high-resolution vision required for activities such as reading, recognizing faces, and driving. When a macular hole develops, central visual acuity is compromised, leading to symptoms such as blurred or distorted vision, central scotoma, and a reduction in color perception. Full-thickness macular defects can hinder visual acuity. Population-based studies have estimated the prevalence of macular holes to range from 0.02% to 0.17% [1–3], with incidence rates ranging from 7.8–8.69 per 100 000 person-years in a Caucasian population [4] up to 30 per 100 000 person-years [3], and 3.14 per 100 000 person-years requiring surgery in a South Korean population [5]. Female sex and increasing age have a higher prevalence [6], incidence [2, 5], and rates of requiring surgery [2, 5].

The goal of macular hole repair is to achieve anatomical closure and subsequent functional improvement. Several surgical techniques have been developed to address macular holes. The gold standard technique is pars plana vitrectomy (PPV) combined with internal limiting membrane (ILM) peeling and gas tamponade. Although most patients respond well to initial treatments, a subset of patients may develop refractory macular holes that require multiple surgeries for closure.

In this case report, we describe the successful closure of a refractory giant macular hole (15 sq mm) with placement of an amniotic membrane graft with improvement in visual acuity. Permission to utilize all medical findings, including images, and informed consent were procured for this case report.

## Case report

A 40-year-old male with a past medical history of HIV, syphilitic retinitis, and vitreoretinal lymphoma (large B-cell lymphoma) with retinal detachment of the right eye (diagnosed at age 34) presented for a second opinion on the management of a refractory macular hole in the right eye. The past surgical history of the right eye included scleral buckle with pars plana vitrectomy, lensectomy, endolaser, and silicone oil placement for the management of retinal detachment at age 34 years. Ten weeks after surgery, he was diagnosed with a macular hole and later underwent an unsuccessful surgical repair attempt with silicone oil removal, membrane peel, and endolaser at the age of 35 years. The patient had no history of surgery in the left eye.

**Figure 1:**
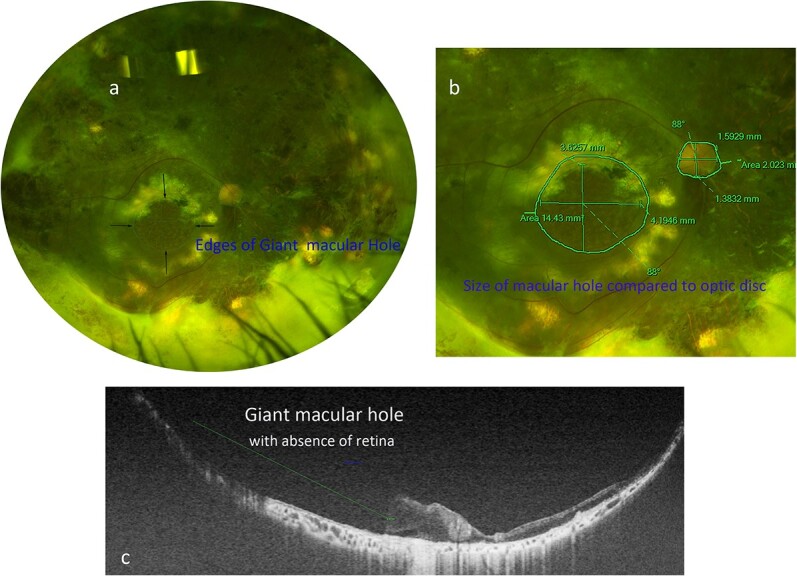
(a) Optos fundus photograph of the right eye showing a large refractory macular hole temporal to the optic nerve. (b) Zoomed-in Optos photograph of the posterior pole demonstrating the measurements of the large macular hole as well as the measurements of the optic nerve for comparison. (c) Swept-source OCT image taken across the diameter of the macular hole, demonstrating its extensive length (green line).

Upon presenting for a second opinion, the patient’s best-corrected distance visual acuity (BCVA) was hand motion in their right eye and 20/20 in left eye. The intraocular pressure was 16 mmHg in the right eye and 20 mmHg in the left eye. The anterior segment examination was remarkable for inferior and superior iridotomies in the right eye and unremarkable in the left eye. Fundus examination of the right eye revealed media opacities, a pale disc with peripapillary elevation due to subretinal fluid, a large macular hole measuring 15 sq mm (Fig 1a and b) with temporal subretinal fluid, and an area of large exudation in the periphery with a visible outline of the scleral buckle and peripheral laser scars amidst areas of fibrosis. Optical coherence tomography (OCT) confirmed the absence of the central retina (Fig 1c). Fundus examination of the left eye revealed vascular attenuation, a small area of inferotemporal chorioretinal atrophy, and two small whitish-colored subretinal lesions.

The patient underwent standard 23-gauge pars plana vitrectomy with careful dissection and removal of the proliferative membranes exerting traction on the giant macular hole. After complete removal of the membranes, an amniotic membrane graft (4 mm × 4 mm) was introduced through sclerotomy and placed into the bed of the macular hole. The edges of the graft were tucked under the rim of the macular, and the graft was secured in place with perfluorocarbon liquid. Endophotocoagulation was then applied to areas of retinal thinning superior to the arcade. The peripheral retina was examined to confirm the absence of additional breaks prior to air-fluid exchange. Additional perfluorocarbon liquid was injected for short-term tamponade and all sclerostomies were sutured closed with 10 ‘0’ nylon sutures.

**Figure 2:**
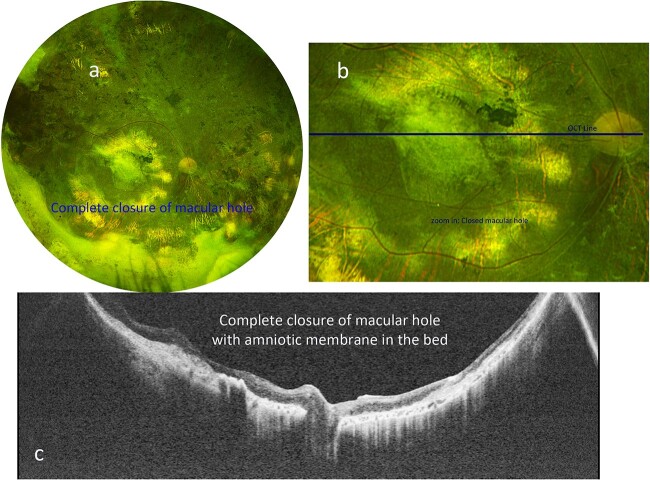
(a) Optos color fundus photograph of the right eye after amniotic membrane placement, with tissue remaining in place above the location of the previously demonstrated macular hole. (b) Zoomed-in Optos color photograph of the posterior pole demonstrating the graft in place. The green line indicates the location of the swept-source OCT in *c*. (c) Swept-source OCT shows the hole has been closed with amniotic membrane graft in place, and flat without subretinal fluid.

One month after surgery, the BCVA in the right eye improved to counting fingers at one foot, and the amniotic membrane graft remained attached in the bed of the macular hole. Four months postoperatively, the patient underwent uneventful removal of perfluorocarbon liquid from the eye. One year after amniotic membrane graft placement, the patient’s BCVA improved to 20/200 in the right eye, with an intraocular pressure of 12 mmHg, and continued attachment of the amniotic membrane graft in the bed of the macular hole (Fig 2a and b). OCT confirmed integration of amniotic membrane graft with complete closure of macular hole (Fig 2c).

## Discussion

While macular holes were initially considered untreatable 30 years ago [7], advancements in diagnostic tools and surgical techniques have revolutionized the management of macular holes, leading to improved outcomes and visual recovery [8, 9]. The exact cause of macular holes remains unclear, but several risk factors have been identified. Age-related changes, vitreous traction, and retinal degeneration are thought to play a crucial role. The predisposing factors include vitreomacular adhesion, posterior vitreous detachment, myopia, trauma, and macular cysts.

The clinical presentation of macular holes involves a variety of visual disturbances. Early stage MHs may present with subtle visual changes, while advanced cases exhibit more profound symptoms. OCT has revolutionized the diagnosis and management of macular holes by providing detailed cross-sectional images of the retinal layers and enabling accurate classification and measurement of the defect [10].

Several surgical techniques have been developed to address macular holes. The gold standard technique is PPV combined with ILM peeling and gas tamponade [9].

Although most patients respond well to initial treatment, a subset of patients may develop refractory macular holes that require multiple surgeries for closure [11]. In particular, patients with larger macular holes (>650 μm) had worse closure rates [8]. For these patients, other approaches, such as the inverted ILM flap technique, macular plug, and autologous neurosensory retinal free flap, have emerged as viable alternatives, particularly for large and refractory holes [12–14]. Autologous retinal grafting with temporary perfluorooctane placement is another promising alternative for patients with refractory macular holes^15^.

Typically, holes measure 100–2000 microns. In our case macular hole measured 15 sq mm (7X the largest described in literature). The improvement in visual acuity in this case with successful closure of such a large refractory macular hole with an amniotic membrane graft demonstrates the utility of the technique in desperate situations.

In summary, we describe successful closure of a refractory giant macular hole with amniotic graft (confirmed on OCT) with improvement in visual acuity.

## Data Availability

Data is available upon request to the corresponding author.
